# A biomechanical study on proximal junctional kyphosis following long-segment posterior spinal fusion

**DOI:** 10.1590/1414-431X20197748

**Published:** 2019-04-25

**Authors:** Wen-Yi Zhu, Lei Zang, Jian Li, Li Guan, Yong Hai

**Affiliations:** Department of Orthopedics, Beijing Chao-Yang Hospital, Capital Medical University, Beijing, China

**Keywords:** Adult spinal deformity, Proximal junctional kyphosis, Upper instrumented vertebra, Functional spinal unit, Finite element

## Abstract

Posterior long-segment spinal fusion may lead to proximal junctional kyphosis (PJK). The present study sought to identify the appropriate fusion levels required in order to prevent PJK using finite element analysis. A finite element model was constructed based on the whole-spine computed tomography findings of a healthy adult. Nine commonly used posterior spinal fusion methods were selected. Stress on the annulus fibrosis fibers, the posterior ligamentous complex, and the vertebrae after various spinal fusions in the upright position were compared. This study was divided into two groups: non-fusion and fusion. In the former, the stress between the T10 and the upper thoracic vertebrae was higher. Comparing thoracic and lumbar segments in the fusion group, the peak stress values of the upper instrumented vertebrae (UIV) were mainly observed in T2 and L2 whilst those of the UIV+1 were observed in T10 and L2. After normalization, the peak stress values of the UIV and UIV+1 were located in T2 and L2. Similarly, the peak stress values of the annulus fibrosus at the upper adjacent level were on T10 and L2 after normalization. However, the peak stress values of the interspinal/supraspinal complex forces were concentrated on T11, T12, and L1 after normalization whilst the peak stress value of the pedicle screw was on T2. Controversy remains over the fusion of T10, and this study simulated testing conditions with gravitational loading only. However, further assessment is needed prior to reaching definitive conclusions.

## Introduction

Adult spinal deformity (ASD) is a common medical disorder in middle-aged and elderly populations with a prevalence rate of 60% (1). ASD occurs in these patients due to lateral and rotational displacements after intervertebral joint laxity and vertebral instability, which are caused by conditions such as intervertebral disc degeneration, intervertebral altitude loss, and vertebral compression fracture. Furthermore, ASD is directly correlated with degeneration and manifests as pain and dysfunction. Surgery is presently the standard treatment for ASD (2) with the most common procedure being posterior spinal fusion with pedicle screws. Nonetheless, long-segmental fusion may lead to stress concentration at both ends of the instrumentation, resulting in proximal junctional kyphosis (PJK), and even proximal junctional failure (PJF).

A recent survey of patients who underwent posterior spinal surgery revealed that the incidence of PJK could be as high as 39% (3,4). The risk factors for this condition have been found to include old age, preoperative sagittal imbalance, combined anterior and posterior spinal fusion, improper fusion, high body mass index (BMI), and osteoporosis. In particular, fusion to the sacrum or pelvis has been recognized as one of its major risk factors. This said, the relationship between the upper instrumented vertebrae (UIV) and PJK remains a subject of debate.

The above-mentioned risk factors have been mostly investigated in retrospective clinical studies and few experimental studies have been performed on cadavers. According to Cahill et al. (5), it is difficult to accurately measure biomechanical parameters on cadavers because these specimens have degraded to biased measurements during repetitive loading, along with advances in biomechanics. Nevertheless, finite element technology is now able to fully simulate mechanical environmental changes in the human body, which has thereby been widely applied in a variety of clinical contexts (6,7).

Through searching databases, such as PubMed, it was found that the finite element model for risk factors of PJK remains in the exploratory phase. Cammarata et al. (4) established a finite element model that simulated the surgical process in six patients who underwent surgery for scoliosis. After the model was confirmed to be effective, it was used to explore the relationship between different surgical strategies and PJK, with a focus on the UIV and functional spinal unit (FSU). In a study of transition rods, Cahill et al. (5) constructed a finite element model (C6-T12) of healthy adult spines. After stress was loaded onto C6, the stress and moment on the thoracic vertebrae were calculated using load and displacement controls; this method, however, was only designed for this section of vertebrae. Similarly, Park et al. (8) developed finite element models of T12 to S1 using the thoracolumbar spines of healthy adults. With the assumption that the range of motion in the spines of patients who had undergone a variety of fusion procedures was comparable to normal spines, they simulated different fusion models. By applying a specific load to T12, they calculated the changes in stresses on different vertebrae and the results were normalized for comparison. In a biomechanical study on the sacrum, Pasha et al. (9) established a finite element model of the spine (from T1 to the pelvis), with the patient's own body weight as the loading condition. Sevrain et al. (10) investigated the effects of different sacral parameters on the lumbosacral junction in patients with spondylolisthesis, in which body weight was also used as the loading condition. Thus, there exist two types of three-dimensional spine models: a simulation based on imaging findings in affected patients (4,9,10) and imaging findings in healthy subjects (5,8). In addition, there were two loading conditions: one was the independent load (5) while the other was the proportion of body weight on the vertebrae (4,8–10). Bess et al. established another finite element model of the spine (from T7 to L5) and the stress and moment on the low thoracic vertebrae were analyzed (11). In spite of the above, no finite element research has described the relationship between fused levels and PJK.

In the present study, the researchers attempted to construct a spinal model (from T1 to the sacrum) in a healthy adult. By loading the subject's own body weight on the vertebrae in the upright position, different models of spinal fusion were constructed. By comparing the results for fusion and non-fusion levels, the distribution of stress in the FSU of the UIV and vertebra above it (UIV+1) were analyzed in an attempt to assist surgeons in selecting the appropriate superior end vertebra.

## Material and Methods

### Patient selection

The present study was approved by the Ethics Committee of Beijing, Chaoyang Hospital, Capital Medical University. A healthy 45-year-old adult male with a body weight of 80 kg was enrolled in the study. The subject was instructed to assume a supine position and a full-length computed tomography (CT) scan with a slice thickness of 10 mm was performed. The files were subsequently imported onto a laptop.

### Construction of geometric models

**Figure 1.**
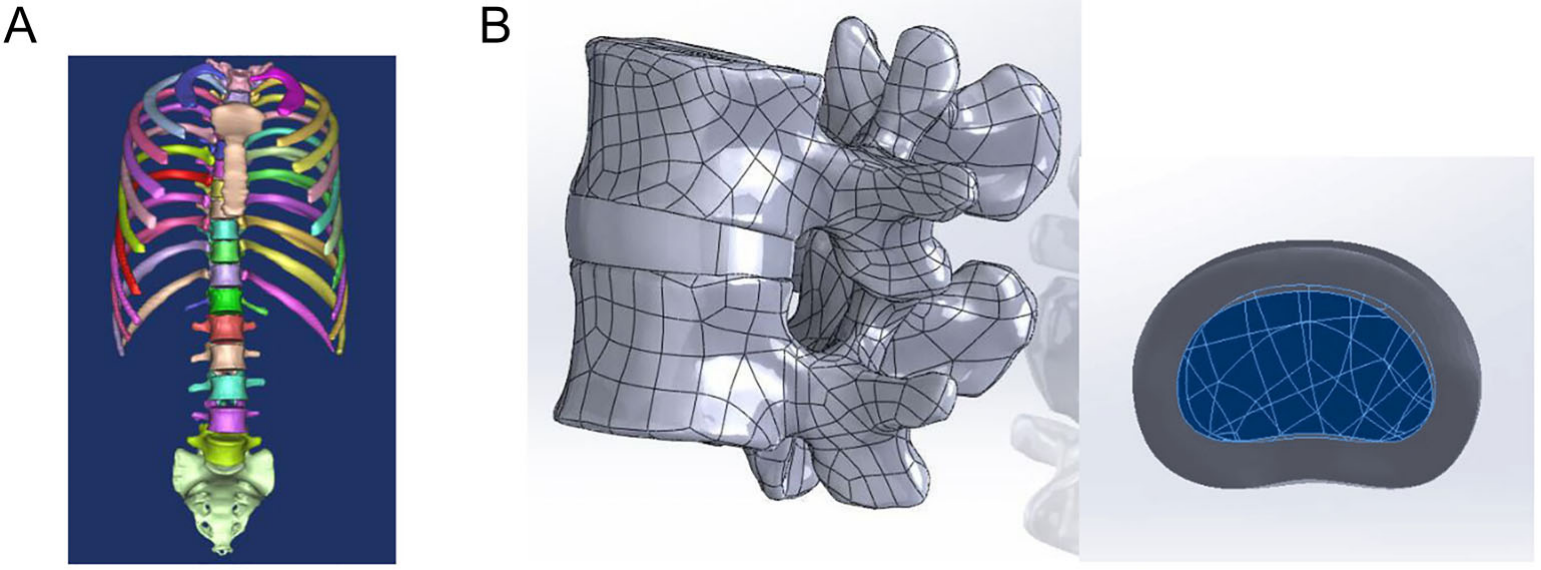
Spinal 3D model. **A**, The whole model, including the T1-S1 vertebrae, chest, and sacrum. **B**, Functional spinal unit and disc.

The CT images were imported into Mimics software (Materialise, Belgium). First, the vertebral bodies were segmented according to the different gray-scale values of the vertebral bones and surrounding tissue. Then, a three-dimensional (3D) reconstruction was performed using the two-dimensional imaging data of the segmented vertebral bodies to produce T1-S1 3D geometric models. Finally, the image of the 3D model was smoothed. Figure 1 presents the reconstructed 3D models of the T1-S1 vertebral bodies. Geometric models of the intervertebral discs, ligaments, and ribs were subsequently constructed.

### Construction of the finite element model

#### Defining the material properties

**Figure 2.**
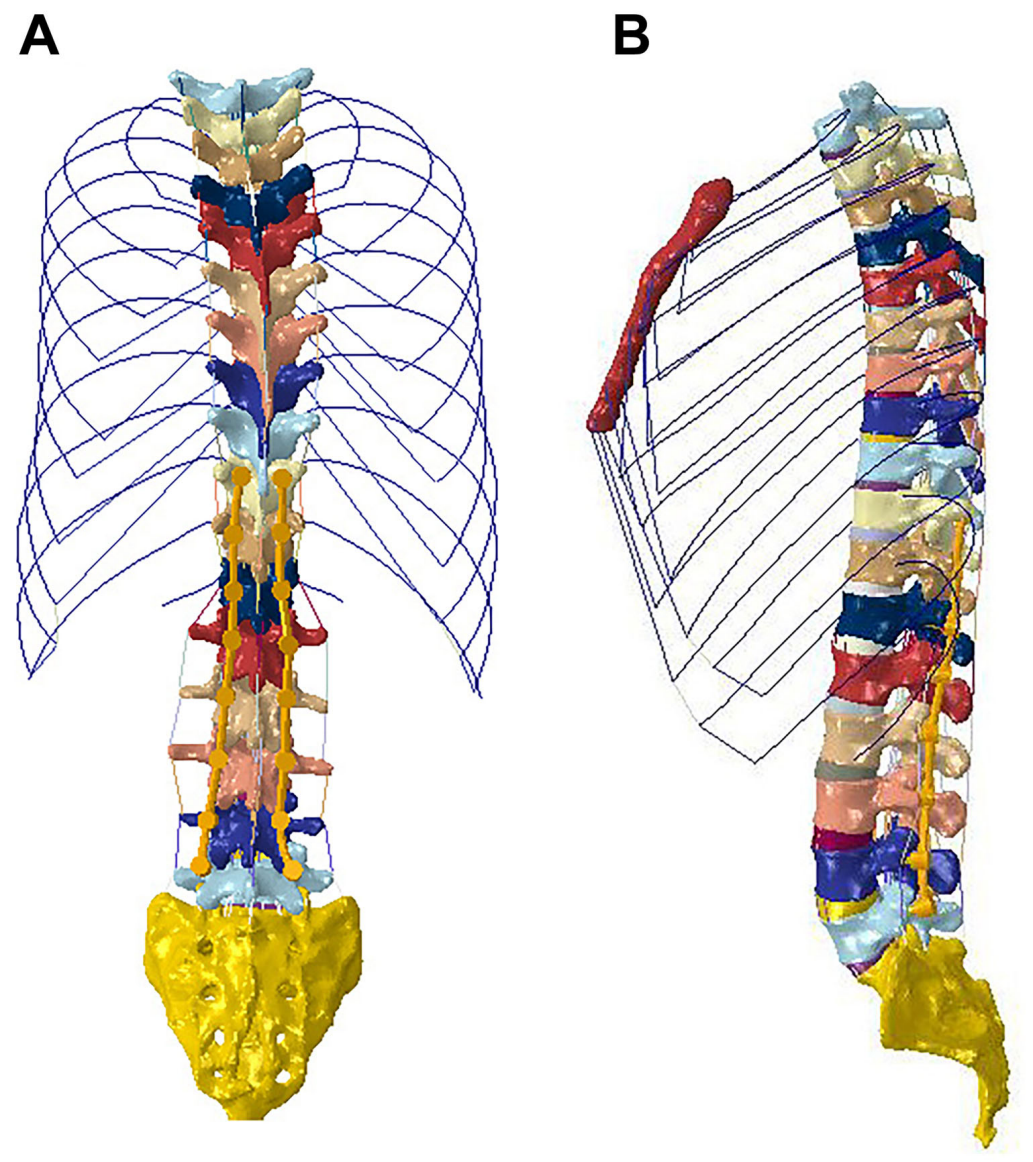
Generation of the finite element mesh. **A**, Front view. **B**, Lateral view.

The model of the segmented vertebral bodies, ligaments (anterior longitudinal, posterior longitudinal, supraspinal (SSL), interspinal (ISL), ligamentum flavum, and intertransverse), intervertebral discs, and ribs were imported into a finite element analytical program (ABAQUS 6.11 Simulia, USA) for simulation and quantitative analysis. Figure 2 presents the finite element model after finite element mesh generation. The physical parameters of the materials were assigned to all the spinal components, as previously described (12,13) (Table 1).

**Table 1.** Material properties used in the model.

Component	Young's modulus (MPa)	Poisson's ratio	Cross-section (mm^2^)
Cortical bone	12,000	0.3	
Cancellous bone	100	0.2	
Endplate	3,000	0.25	
Anterior longitudinal	15		40
Posterior longitudinal	10		20
Ligamentum flavum	8		30
Interspinous	10		40
Ligamentum flavum	15		40
Intertransverse	10		1.8
Capsular	7.5		30
Nucleus pulposus	1.0	0.499	
Annulus fiber	4.2	0.45	
Fusion mass (Ti)	110,000	0.28	

#### Defining the boundary and loading conditions

Gravitational forces were modeled for each vertebra. These were craniocaudal forces with magnitudes specifically proportional to body weight, as previously described (4,14) (Table 2). The body weight of the subject was 80 kg; hence, the gravitational acceleration was 9.8 m/s^2^. A tie constraint was applied to simulate the junctions between vertebral bodies and intervertebral discs and those between vertebrae and ligaments, allowing the contact surfaces between the vertebral bodies and intervertebral discs as well as the junctions between the vertebrae and ligaments to remain unseparated under force. The lower parts of the sacrum were also fixed and the tie constraint was used in the junctions between the vertebral bodies and pedicle screws.

**Table 2.** Gravitational properties at each vertebral level.

Level	Load (patients' weight, %)	Strength (N; weight: 80 kg)
Head + arms + T1	20.9	163.9
T2	1.1	8.6
T3	1.4	11.0
T4	1.3	10.2
T5	1.3	10.2
T6	1.3	10.2
T7	1.4	11.0
T8	1.5	11.8
T9	1.6	12.5
T10	2.0	15.7
T11	2.1	16.5
T12	2.5	19.6
L1	2.4	18.8
L2	2.4	18.8
L3	2.3	18.0
L4	2.6	20.4
L5	2.6	20.4

#### Simulation of different fusion conditions

Based on the posterior spinal fusion approach using pedicle screws, it was assumed that the bottom of the instrumentation would be at L5 because it is known that the PJK increases due to fusion with the sacrum. In order to further investigate this, the following nine fusion segments were selected: L4-L5, L3-L5, L2-L5, L1-L5, T12-L5, T11-L5, T10-L5, T4-L5, and T2-L5 and all pedicles within these segments were fixed with screws. The biomechanical changes in the FSU of the UIV and UIV+1 were studied using the finite element model. Different vertebral bodies had different spatial positions and the distribution of stress that these carried also differed. Thus, it was impossible to compare the stress on different segments. The normalization principle (8) was used in the present study; that is, changes in stress at the same level before and after fusion were compared.

## Results

The PJK was associated with the FSU of the UIV and UIV+1. The distribution of stress in the nine different models was principally investigated by simulating the UIV, the superior fibrous ring, and the UIV+1 in an upright position. Thus, a total of ten independent models were constructed, which included one non-fusion model in addition to the nine fusion models.

### Stress distribution in non-fusion models in the upright position

**Figure 3.**
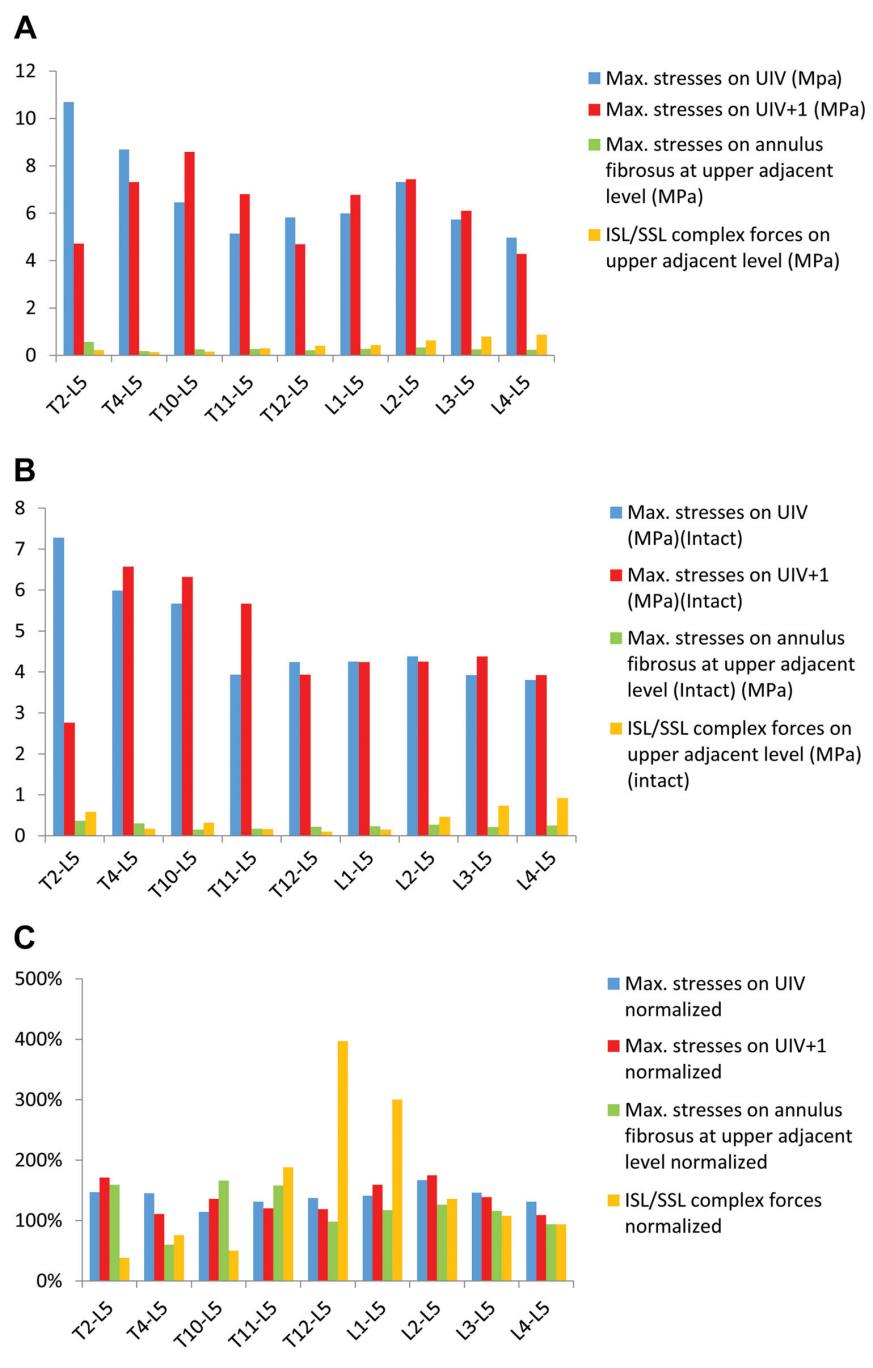
Selection of nine types of fusion models and a comparison of the maximal von Mises stresses of the UIV, UIV+1, and both the annulus fibrosus and the ISL/SSL at the upper adjacent level in the fusion model (**A**), intact model (**B**), and normalized model (**C**). UIV: upper instrumented vertebrae; SSL: supraspinal: ISL: interspinal.

**Figure 4.**
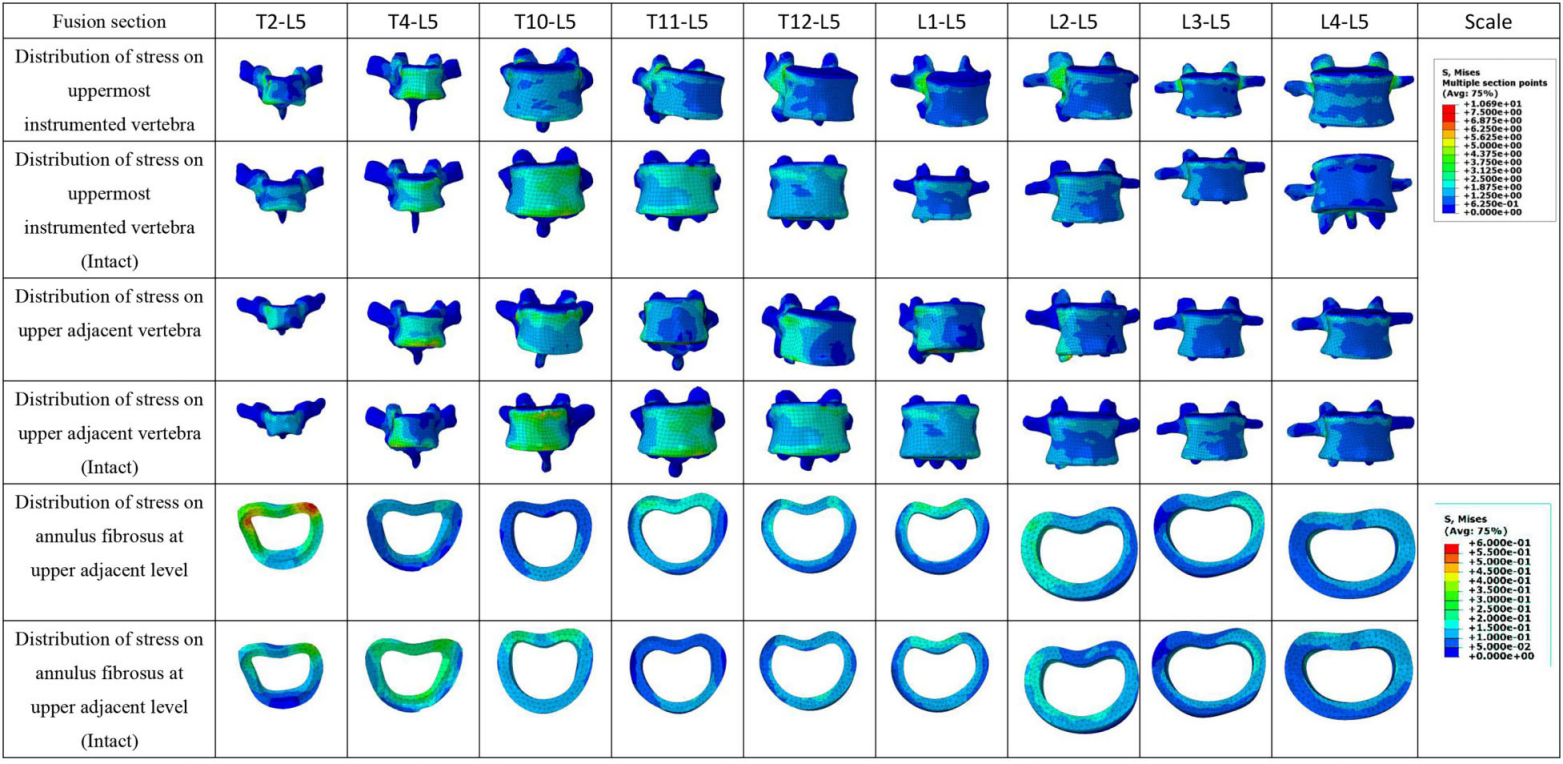
The distribution of stresses on the upper instrumented vertebrae (UIV), UIV+1, and the annulus fibrosus at the upper adjacent level in the fusion model as well as comparisons with the same level in the intact model.

The maximum von Mises stress in different vertebrae ranged from 3.8 to 7.28 MPa (mean±SD: 4.8±1.14 MPa). Compared with the stresses on the fibrous rings that ranged from 0.15 to 0.36 MPa (mean±SD: 0.24±0.06) and the ISL/SSL complex forces on the upper adjacent level that ranged from 0.10 to 0.92 MPa (mean±SD: 0.4±0.29 MPa), the stresses on the vertebrae were ≥10 times higher. Furthermore, the changes in stress were more obvious from T10 to the upper thoracic segments (Figure 3), whilst the stresses on the lower thoracic segments were mainly concentrated at the front of the vertebrae. (Figure 4).

### Stress distribution in fusion models in the upright position

The maximum von Mises stresses on the UIV ranged from 4.96 to 10.69 MPa (mean±SD: 6±0.76 MPa), significantly higher than those in the non-fusion models. In these differing stress patterns, there were two peaks at the thoracic and lumbar segments, respectively. One peak value was detected on L2, which reached 7.41 MPa, and another peak value was detected on T2, which reached 10.69 MPa. The maximum von Mises stress in the UIV+1 ranged from 4.28 to 8.58 MPa (mean±SD: 6.17±1.41 MPa). Similarly, two peak values were detected: one on the UIV+1, which reached 6.1 MPa when the UIV was fused to L2; and one on the UIV+1, which reached 8.59 MPa when the UIV was fused to T10. The ISL/SSL complex forces on the upper adjacent level ranged from 0.16 to 0.87 MPa (mean±SD: 0.44±0.27 MPa). The maximum stresses on the annulus fibrosus at the upper adjacent level ranged from 0.21 to 0.57 MPa (mean±SD: 0.28±0.11 MPa). After fusion, all stresses on the UIV were concentrated on the rear of the vertebrae (Figure 4).

In addition, it was observed that the maximum von Mises stress was on the pedicle screw of the UIV. Accordingly, double-sided pedicle screw forces were measured and averaged at each level (Table 3). It was found that the maximum von Mises stress of the screw force increased dramatically when fixed from L5 to T2.

**Table 3.** Selection of 9 fusion models and comparison of the maximal von Mises stresses on the pedicle screw.

Fusion model	Max. screw stress (MPa)
T2-L5 fusion	106.50
T4-L5 fusion	48.14
T10-L5 fusion	45.50
T11-L5 fusion	44.68
T12-L5 fusion	42.66
L1-L5 fusion	49.97
L2-L5 fusion	48.71
L3-L5 fusion	47.59
L4-L5 fusion	55.22

### Normalization results of the FSU, UIV, and UIV+1

The stress on the UIV was normalized before and after fusion and two stress peaks were observed: as high as 167% when fused to L2 and 147% when fused to T2. In addition, two peak values were observed on the UIV+1 of 175% when the UIV was fused to L2 and 171% when the UIV was fused to T2. The peak values of stresses on the adjacent fibrous rings were observed when the UIV was fused to T11, T10, and T2, attaining 158, 166, and 159%, respectively (Table 4, Figure 3). Nevertheless, the normalization results for the ISL/SSL complex forces on the area of the thoracolumbar junction were the highest compared to other vertebrae. The normalization of the ISL/SSL complex forces reached 188% on T11, 397% on T12, and 300% on L1 (Table 4, Figure 3).

**Table 4.** Selection of 9 fusion models and comparison of the maximal von Mises stresses among UIV, UIV+1, annulus fibrosus at upper adjacent level, and ISL/SSL on upper adjacent intervertebral space in the fusion model, intact model, and normalized.

Fusion model	T2-L5	T4-L5	T10-L5	T11-L5	T12-L5	L1-L5	L2-L5	L3-L5	L4-L5
Max. stress on UIV (Mpa)	10.69	8.69	6.46	5.14	5.82	5.99	7.32	5.73	4.96
Max. stress on UIV (MPa) (Intact)	7.28	5.99	5.67	3.93	4.24	4.25	4.38	3.92	3.8
Max. stress on UIV normalized (%)	147	145	114	131	137	141	167	146	131
Max. stress on UIV+1(MPa)	4.71	7.31	8.59	6.81	4.69	6.77	7.43	6.1	4.28
Max. stress on UIV+1 (MPa) (Intact)	2.76	6.57	6.32	5.67	3.93	4.24	4.25	4.38	3.92
Max. stress on UIV+1 normalized (%)	171	111	136	120	119	159	175	139	109
Max. stress on annulus fibrosus at upper adjacent level (MPa)	0.57	0.18	0.25	0.26	0.21	0.27	0.33	0.27	0.23
Max. stress on annulus fibrosus at upper adjacent level (Intact) (MPa)	0.36	0.30	0.15	0.17	0.22	0.23	0.27	0.21	0.25
Max. stress on annulus fibrosus at upper adjacent level normalized (%)	159	60	166	158	98	117	126	116	94
ISL/SSL complex forces on upper adjacent level (MPa)	0.22	0.13	0.16	0.30	0.40	0.44	0.63	0.79	0.87
ISL/SSL complex forces on upper adjacent level (MPa) (intact)	0.58	0.17	0.32	0.16	0.10	0.15	0.46	0.73	0.92
ISL/SSL complex forces normalized (%)	38	76	50	188	397	300	136	108	94

UIV: upper instrumented vertebrae; ISL: interspinal; SSL: supraspinal.

## Discussion

It is imperative that spinal coronal and sagittal imbalances be considered during the surgical treatment of ASD. Long-level fusion is often required, which creates a concentration of stress at both ends of the instrumentation and may lead to junctional kyphosis, especially PJK, or PJF. Clinical research has demonstrated a close relationship between the fusion location of the proximal vertebrae and the development of PJK.

PJK and PJF mainly occur at the proximal end of the instrumentation. The development of the former is closely associated with the degeneration or injury of intervertebral discs, which is mainly caused by stress variations on the fibrous rings. The principal cause of PJF, however, is compression fracture at the UIV/UIV+1 level. Consequently, determining stress variations on the UIV, UIV+1, and intervertebral discs is the most common method of studying PJK and PJF.

In the present study, the biomechanical effects of the fusion level and its role in proximal junctional problems, including PJK and PJF, were investigated by constructing a computer model of the section from T1 to the sacrum. Finite element (FE) models of nine different spinal fusions were developed using a validated FE model of a healthy spine. Several biomechanical properties, including stress on the annulus fibrosus fibers, posterior ligamentous complex, and the UIV/UIV+1 level with and without pedicle screws were compared and analyzed.

In the present study, it was considered that different vertebrae have different spatial locations and different areas of stress concentration. As such, it was problematic to compare the absolute values of the stresses in these areas, even though previous experiments have cited this as a solution for normalizing the values of stresses in fusion and non-fusion states for the purposes of comparison. The stress on the UIV and its adjacent vertebrae was higher from T10 to the upper thoracic segments than in the lumbar segments prior to the pedicle being fixed, which may be explained by the local anatomy; this is the transitional region between the lumbar lordosis and thoracic kyphosis, where T11 and T12 (as floating ribs) have the greatest mobility. In contrast, the tenth rib is relatively fixed and the connection between T10 and the thoracic cage is more stable. Therefore, T10 represents a stress concentration level.

After spinal fusion with the pedicle screw, it was found that there were two peak values in the lumbar and thoracic segments: one was T2 and the other was L2. It was evident that careful selection should be made when fusing these two levels.

When the location of the UIV is at the thoracic level, there is controversy as to whether the upper or lower thoracic level should be used. Smith et al. (15) found that the incidence of PJK was lowest when fusion was performed on the upper thoracic segments. Similarly, Scheer et al. (16) demonstrated that in patients who had undergone pedicle subtraction osteotomy, fusion to the upper thoracic vertebrae (T1-T6) was associated with a lower incidence of PJK than when fusion took place in the lower vertebrae (T9-L1). Diebo et al. (17) also argued that UIV should be at T2-T3. However, Arlet and Aebi (18) claimed that fusion at T1-T3 represented an independent risk factor for PJK. Meanwhile, Zhu et al. (19) suggested that long-level fusion to T11 and T12 was a more reasonable option whilst more recent studies (20–22) have indicated that fusion to both the upper and lower thoracic vertebrae are associated with comparable incidences of PJK. Nevertheless, fusion to the lower thoracic vertebrae are associated with a high risk of fractures whereas fusion to the upper thoracic vertebrae were more likely to cause dislocation. In the present study, however, when the fusion level was T2, changes in stresses at the FSU of the UIV/UIV+1 segment reached a peak value (UIV: 147%; UIV+1: 171%; and 159% at the fibrous rings), but the lowest value was 38% (at the ISL/SSL complex forces). Therefore, it was considered that fusion to T2 was linked to higher risk of PJK and fractures. In contrast, the stress on the vertebrae may be lower when the UIV is at the lower thoracic levels, meaning a lower risk of PJK and fractures. However, the maximal von Mises stress values of the ISL/SSL complex forces on the lower thoracic levels were higher than those on the upper thoracic levels. Thus, fusion to lower thoracic levels carries a higher risk of dislocation than fusion to upper thoracic segments. In addition, it was found that the pedicle screw force was greatest when the long spinal fusion equipment was fixed to T2 than when it was fixed to other levels. Hence, a fusion level at T2 was not recommended.

When fused to T2 and T4, the normalized values of the UIV (147 and 145%) were similar, but there was a significant difference when compared to the UIV+1 (171 and 111%). In addition, when fused to T4, the normalized values of the annulus fibrosus was the lowest (60%) and the ISL/SSL complex force was lower (76%) compared to when fused to other thoracic levels, revealing that the risk of damage to the annulus fibrosus was lower. Consequently, the study concluded that fusion to T4 should be preferable to T2.

The UIV at the lower thoracic levels also presented different options. According to Shufflebarger et al. (23), since T11 and T12 are floating ribs, these should be crossed over and the fusion placed at T10 or the vertebrae above it. However, Cho et al. (24) found similar levels of effectiveness between fusion to T10 and fusion to T11 or T12. In the present study, after normalization, the ISL/SSL complex force was lower (50%) when the UIV was selected at T10. In contrast, the ISL/SSL complex forces (188, 390%) were highest when the UIV was at T11 and T12, along with a higher risk of dislocation. In addition, the stress of the ISL/SSL complex forces at L1 was 300% and the posterior ligament complex exhibited the greatest stress change in the thoracolumbar junction area. It was possible that this region was the transition from the movable lumbar to the relatively fixed thoracic levels; thus, stress was more concentrated here and it was obviously deemed inappropriate to fuse to the thoracolumbar junction area.

This study showed that the normalized UIV was the lowest value (114%) while the ISL/SSL complex forces were lower (50%) when the UIV was selected at T10; thus, it seemed that fusion to T10 was the better choice. However, the stress on the annulus fibrosus was the highest (166%) at T10 so its risk of damage was greater and would accelerate intervertebral disc degeneration. Hence, the notion of fusing at T10 remains controversial.

For fusion at the lumbar levels, controversy also remains over whether the UIV should be at L1 or L2. In 1999, Lee et al. (25) proposed that UIV at L2 was a risk factor for PJK and a study conducted by Zhu et al. (19) revealed that fusion at L1 or L2 had the highest incidence of PJK. However, Arlet and Aebi (18) argued that the UIV should be at L1, T12, and T11. Furthermore, Kim et al. (26) divided a group of 125 patients with adult degenerative spinal deformity into three groups: T9-T10, T11-T12, and L1-L2. The average follow-up period was 4.5 years, during which fusion to L1-L2 showed the same clinical effectiveness as fusion to the thoracic vertebrae. In the present study, when the fusion level was at L2, the maximum von Mises stresses on the UIV, UIV+1, and the FSU reached a peak whilst the normalized values also peaked (UIV: 175%; adjacent fibrous ring: 126%; UIV+1: 175%; and ISL/SSL complex force: 136%). When the fusion level was at L1, the normalized value of the ISL/SSL complex force (300%) was highest. As such, it was speculated that UIV fusion to L1 and L2 is associated with a higher risk of PJK.

The present study had some limitations. First, PJK has been shown to increase due to fusion with the sacrum so the investigators purposely avoided this section, as it would lead to significant stress changes. This is to be discussed in subsequent studies. Second, the investigators constructed a finite element analysis model in a healthy adult and only compared the stress distribution on the sagittal plane (though, in fact, coronal imbalance is also common in ASD patients). Third, in all samples, the stresses were compared in the upright position but not in daily activities. Finally, the investigators avoided examining the biomechanical impact of the muscles during the construction of the experimental models, which might have affected the accuracy of the measurement results.
